# Development of a shared item repository for progress testing in veterinary education

**DOI:** 10.3389/fvets.2023.1296514

**Published:** 2023-11-02

**Authors:** Elisabeth Schaper, Theo van Haeften, Jakob Wandall, Antti Iivanainen, Johanna Penell, Charles McLean Press, Pierre Lekeux, Peter Holm

**Affiliations:** ^1^Centre for E-learning, Didactics and Educational Research, University of Veterinary Medicine Hannover, Foundation, Hannover, Germany; ^2^Department of Biomolecular Health Sciences, Faculty of Veterinary Medicine & Centre for Academic Teaching and Learning, Utrecht University, Utrecht, Netherlands; ^3^NordicMetrics Aps, Copenhagen, Denmark; ^4^Department of Veterinary and Animal Sciences, Faculty of Health and Medical Sciences, University of Copenhagen, Frederiksberg, Denmark; ^5^Department of Veterinary Biosciences, Faculty of Veterinary Medicine, University of Helsinki, Helsinki, Finland; ^6^Department of Clinical Sciences, Faculty of Veterinary Medicine and Animal Science, Swedish University of Agricultural Sciences, Uppsala, Sweden; ^7^Department of Preclinical Sciences and Pathology, Faculty of Veterinary Medicine, Norwegian University of Life Sciences, Aas, Norway; ^8^European Association of Establishments for Veterinary Education (EAEVE), Vienna, Austria

**Keywords:** Progress testing, European veterinary education, veterinary item repository, quality assurance, item writing, day one competencies, adaptive testing, linear testing

## Abstract

**Introduction:**

Progress testing in education is an assessment principle for the measurement of students’ progress over time, e.g., from start to graduation. Progress testing offers valid longitudinal formative measurement of the growth in the cognitive skills of the individual students within the subjects of the test as well as a tool for educators to monitor potential educational gaps and mismatches within the curriculum in relation to the basic veterinary learning outcomes.

**Methods:**

Six veterinary educational establishments in Denmark, Finland, Germany (Hannover), the Netherlands, Norway, and Sweden established in cooperation with the European Association of Establishments for Veterinary Education (EAEVE) a common veterinary item repository that can be used for progress testing in European Veterinary Education Establishments (VEEs), linear as well as computer adaptive, covering the EAEVE veterinary subjects and theoretical “Day One Competencies.” First, a blueprint was created, suitable item formats were identified, and a quality assurance process for reviewing and approving items was established. The items were trialed to create a database of validated and calibrated items, and the responses were subsequently psychometrically analyzed according to Modern Test Theory.

**Results:**

In total, 1,836 items were submitted of which 1,342 were approved by the reviewers for trial testing. 1,119 students from all study years and all partners VEEs participated in one or more of six item trials, and 1,948 responses were collected. Responses were analyzed using Rasch Modeling (analysis of item-fit, differential item function, item-response characteristics). A total of 821 calibrated items of various difficulty levels matching the veterinary students’ abilities and covering the veterinary knowledge domains have been banked.

**Discussion:**

The item bank is now ready to be used for formative progress testing in European veterinary education. This paper presents and discusses possible pitfalls, problems, and solutions when establishing an international veterinary progress test.

## 1. Introduction

Establishing academic progress in students is the main objective for most educational institutions. Progress in knowledge and skills can thus be defined as the main outcome of education when the final outcomes have been met. It has been attempted to measure progress in students reliably for more than 100 years (1). Defining and verifying students’ progress requires measurement of their ability (knowledge, skills, competencies, etc.), which is expected to develop during studying and training. Progress can be defined as the difference between two consecutive test scores of the same student, measured on the same scale. In this context, a Progress Test is defined as a test that is designed for longitudinal measurement of educational progress for learning and formative purposes.

Progress testing in health science education is an assessment principle for the measurement of students’ progress from study start to graduation (2). It was introduced simultaneously in Europe and North America in the 1980s by the medical faculties of Maastricht University and Kansas City University (2, 3). It has since been adopted in medical programs all over the world (4–7) including veterinary medicine (8, 9). Progress testing is applied as a series of multiple-choice tests (1–4 per year) throughout the study. Each test usually consists of 100–200 items covering all content areas of the curriculum and targets the academic level expected by the students at graduation within all subjects of the curriculum (10, 11).

Usually, the underlying aim of progress testing is to stimulate longitudinal and lasting learning by supplying students with frequent individual feedback on their progress, strengths, and weaknesses in knowledge and competencies within the content of the curriculum (11). At the same time, progress testing may serve as a quality assurance tool for educators allowing the identification of curricular progress, strengths, and weaknesses in student cohorts, and possibly also of individual students during their study. Finally, progress testing may be used as a tool for benchmarking to a common standard, if tests are organized as a collaborative effort between identical education programs drawing on test items that have been psychometrically validated across these institutions (11–13).

Progress testing is generally implemented as a low-stakes test for formative reasons or for combined formative and summative purposes. At some faculties, students’ engagement is voluntary, at others, taking the tests is compulsory but results are only used for formative purposes or – as at some medical faculties – progress testing is integrated into their cumulative assessment program, e.g., as part of students’ portfolios (10).

As in most other assessment systems, the instruments (the tests and the items) in progress testing are expected to be valid, meaning that they measure the construct (construct validity) and cover the content they are supposed to assess (content validity). Furthermore, the measures (scores) of the student’s performance must be reliable and stable (test reliability) (14). Adequate content validity is generally ensured through test blueprinting determining formats, content, and taxonomic levels of the test items (15), and psychometric analyses of test results and item responses are carried out to elucidate if the test produces valid and reliable measures of students’ proficiency within the domain of the test (16).

When establishing a transnational progress test, ensuring that the test results are both accurate and comparable is challenging, as the test is supposed to measure students’ proficiency equally well at all universities and across all study years. The test should function homogeneously despite differences, such as in curricula and language (5). A test blueprint, including well-described and highly standardized item writing guidelines and a clear plan for how to psychometrically assess the responses, is therefore critical as in most assessments (16). Especially when tests involve and utilize a very large number of items like progress testing usually does. This is necessary to avoid fluctuating item quality, which may affect the general test quality and parameter estimates and impair the validity and reliability of the test and the measures (14). Rigorous quality assurance systems need to be implemented, including pre-assessment reviews of items by colleagues and students, and strict psychometric analysis of item responses must be used to estimate the reliability of the scores, how well the item content targets the students’ abilities, identify poor-performing items, and estimate of item difficulty, etc. (14, 16).

In recent years, computer-adaptive progress testing has replaced classical linear progress testing at several medical faculties. In linear testing, all students respond to identical sets of items at each test. In computer adaptive testing, students are presented with items that are selected dynamically from a pool of calibrated items with known (pre-assessed) difficulty. Item selection is done by an algorithm that – based on the student’s ability estimated from same student’s previous responses in the test – selects the next item with a matching difficulty level. Thus computer-adaptive progress testing allows the dynamic adaptation of the test difficulty to the performance of the individual student (17, 18). By doing this, computer adaptive testing circumvents the potential mismatch between students’ abilities and the difficulty level of a test. Such a mismatch is often seen in classical linear progress testing when the test that covers all content domains of a whole curriculum is administered to, e.g., first-year students. A significant discrepancy between test difficulty and the student’s academic level is undesirable as it may discourage some students, affect the reliability of the test result, and may lead to unintended study behavior (19). Computer-adaptive testing can achieve robust reliability in test measures while utilizing just 50% of the items required in a traditional linear test (18), which means reduced test time for students. This is advantageous for maintaining students’ concentration and engagement during repeated tests.

In linear progress testing, the analysis of test scores, including estimation of test difficulty has traditionally relied on Classical Test Theory and has been conducted post-testing, often without prior trailing of items. Computer-adaptive testing relies on trialing (trial-testing) of all items and psychometric analyses using Modern Test Theory, specifically Item Response Theory (IRT) or Rasch Modeling (14, 20).

In 2020, six veterinary educational establishments (VEEs) from six EU Countries and the European Association of Establishments for Veterinary Education (EAEVE) established a collaboration with the purpose of creating a common English-language veterinary item repository complying with the Annex III of the EU Directive on the recognition of professional qualifications (21) and covering the veterinary subjects and theoretical Day One Competencies approved by EAEVE and the European Committee of Veterinary Education (ESEVT) (22). The idea was that the item repository could serve as a shared educational instrument for all European VEEs, facilitating both linear and computer-adaptive progress testing across countries and veterinary educational institutions. Given the resource-intensive and costly nature of establishing such an item repository, an application for a 3-year project was submitted to the EU Erasmus Plus program and was granted in August 2020 (23). The present study aims to describe the process leading to the establishment of a validated item repository.

We will discuss and evaluate the process with respect to (i) test blueprinting, including item formats, (ii) quality assurance procedures for pre-assessment of items, item writing guidelines and materials, (iii) item writing, (iv) item trialing, (v) psychometric validation of items using Modern Test Theory (the Rasch Model), and (iv) the perspectives of implementation of common veterinary progress testing drawing on the established item repository.

## 2. Methods

### 2.1. Formation of the VetRepos collaboration

Initially, senior veterinary educators from the six veterinary educational establishments (VEEs) in Denmark, Finland, Hannover (Germany), the Netherlands, Norway, and Sweden [all members of the European Association of Establishments for Veterinary Education (EAEVE)] met in June 2019 to discuss the possibilities for the establishment of a common veterinary item repository that could be used for progress testing, linear as well as computer adaptive, across European VEE’s. The EAEVE was invited into the collaboration, as one of its objectives is to reinforce cooperation between member establishments to improve and harmonize the measurement of outcomes of veterinary education (24).

Four of the VEEs had no history with progress testing but expressed their intention to integrate formative progress testing into their future curricula. The Dutch VEE had practical experience with progress testing in their master program (9) and wanted to develop it further to include the bachelor program and implement it as computer adaptive progress testing. The German VEE had extensive experience with linear progress testing (25) and wanted to implement computer-adaptive progress testing. Construction of multiple-choice questions (MCQ) for formative and summative purposes and classroom quizzes were well-known at all VEEs. Experiences with standardized procedures for reviewing MCQ and tests varied, ranging from no standardized procedure at all to supplying guidelines for item writers and requiring peer-review of items by colleagues, external examiners, or review by an exam committee.

### 2.2. Blueprinting of item repository

As a first project step, the partners decided on a one-dimensional blueprint, partly based on the German-speaking VEEs’ experiences (10). The blueprint covers the 34 veterinary science disciplines (subject areas) listed in EAEVE/ESEVT Standard Operational Procedures, Annex 2 (22). VEEs affiliated with EAEVE can relate to the blueprint independently of their type of curriculum. It was also agreed to group the disciplinary items into four veterinary knowledge domains (test subscales) equivalent to the EAEVE/ESEVT veterinary science domains: (i) Basic Sciences, (ii) Clinical Sciences within companion animals and equines, (iii) Clinical Sciences within production animals including Animal production subjects, and (iv) Food Safety Veterinary Public Health and One Health. The EAEVE/ESEVT domain of basic science subjects (chemistry, medical physics, feed plant biology, etc.) was left out since these subjects are not part of all curricula at European VEEs.

The partners anticipated that it would be possible to bank around 800 to 1,200 calibrated items in total during the 3-year project period, allocated as ≈ 600 (50%) items in subscale 1, ≈ 240 (20%) items in both subscales 2 and 3, and ≈ 120 (10%) items in subscale 4, and with an even disciplinary distribution of respective items within the relevant subscales.

Table 1 shows the agreed blueprint with the aimed distribution of items within subscales and disciplines.

**Table 1:** Blueprint for the VetRepos item repository for progress testing.

Test subscales	Veterinary disciplines	Aimed no. of items in subscale (% of total items)	Aimed no. of items per discipline
Basic Sciences (Subscale 1)	17 disciplines: Anatomy, histology and embryology; Physiology; Cell biology, Biochemistry; General and molecular genetics; Pharmacology, pharmacy and pharmacotherapy; Pathology; Toxicology; Parasitology; Microbiology; Immunology; Epidemiology; Information literacy and data management; Professional ethics and communication; Animal health economics and practice management; Animal ethology; Animal welfare; Animal nutrition	600 (50%)	35
Clinical Sciences – Companion Animal & Equine (Subscale 2)	10 disciplines: Obstetrics, reproduction and reproductive disorders; Diagnostic pathology; Medicine; Surgery; Anesthesiology; Clinical practical training; Preventive medicine; Diagnostic imaging; Therapy; Propaedeutics.	240 (20%)	24
Clinical Sciences – Production Animals, including animal production subjects (Subscale 3)	12 disciplines: Obstetrics, reproduction and reproductive disorders; Diagnostic pathology; Medicine; Surgery; Anesthesiology; Clinical practical training; Preventive medicine; Diagnostic imaging; Therapy; Propaedeutics; Animal production, including breeding, husbandry and economics; Herd health management	240 (20%)	20
Food Safety & Quality, Public Health and One Health Concepts (Subscale 4)	5 disciplines: Veterinary legislation including official controls, regulatory veterinary services, forensic veterinary medicine and certification; Control of food, feed and animal by-products; Zoonoses; Food hygiene and food microbiology; Food technology	120 (10%)	24

### 2.3. Item formats

**Figure 1:**
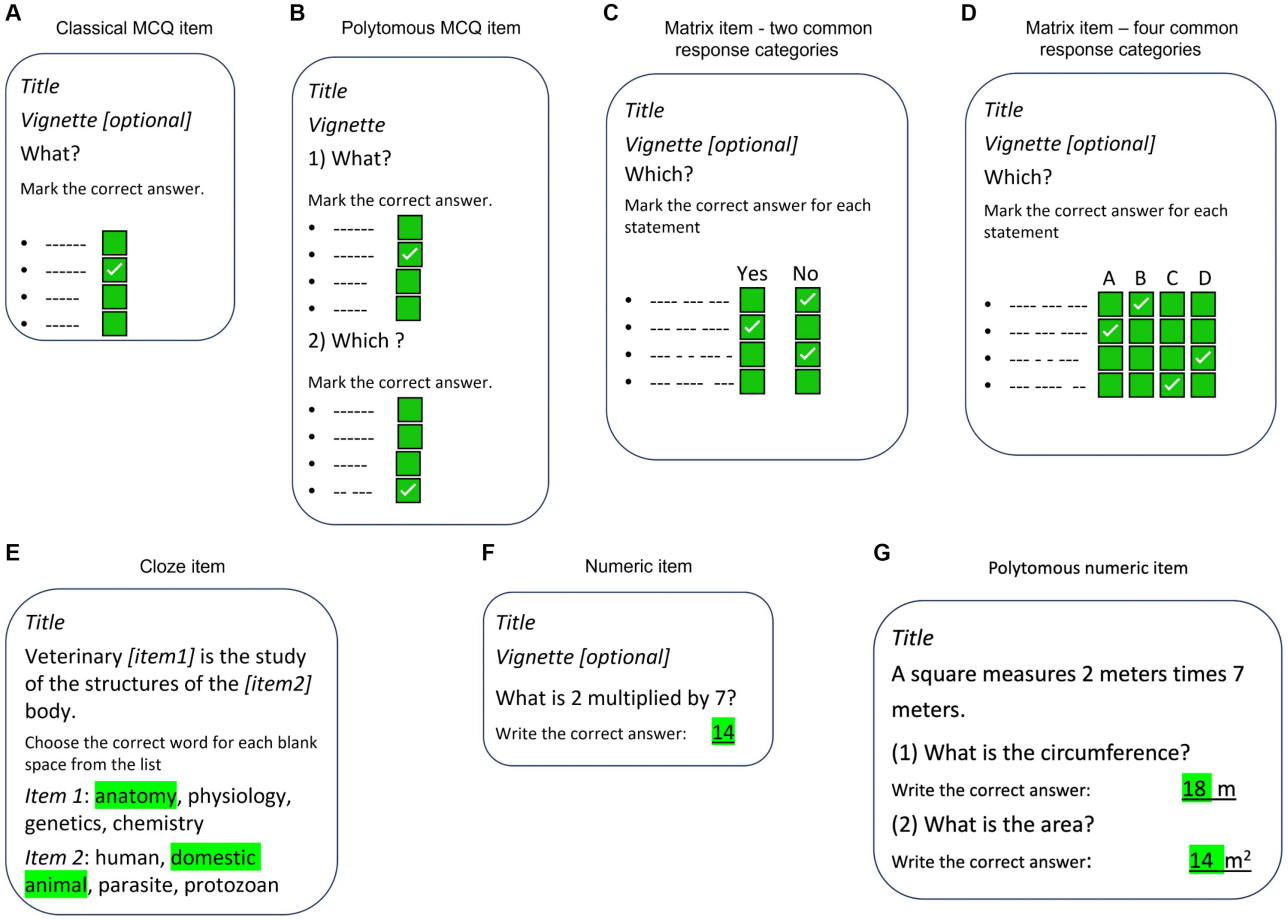
Schematic illustration of the item formats in VetRepos. **(A)** Classical MCQ item format, **(B)** Polytomous MCQ item format, **(C)** Matrix “yes-no” / “true-false” item format, **(D)** Matrix item format with more common response categories, **(E)** Cloze item format, **(F)** Numeric item format, G: Polytomous numeric item format. All item formats may include illustrations/photos in the vignette, and related source information.

The collaboration agreed on six different item formats to be used, of which four are close-ended formats and two open-ended formats: (i) the classical dichotomous MCQ with four response categories (1 correct answer plus three distractors), (ii) a polytomous version of MCQ, in which two or more MCQs are grouped using the same vignette, (iii) the polytomous cloze format with four response categories for each question (item) within its text (one correct word and three distractors), (iv) the matrix format with up to eight questions (subitems) with two to six common response categories (including the true/false and yes/no versions), (v) a numeric response question (NRQ) format where the correct answer is an integer or well-defined decimal number, and finally (vi) a polytomous version of NRQ, in which two or more items are grouped using the same vignette. These item formats are all simple and efficient (26) and cover the projects’ needs. Illustrations were allowed as part of the vignette in all item formats. Moreover, each item was associated with one or more of the four subscales (knowledge domains) and one or more of the 34 veterinary disciplines and labeled with a unique item number, ID of author and university, filename of illustration (if illustration is present), and reference to the source of information (optional). A schematic illustration of the item formats is presented in Figure 1.

### 2.4. The quality assurance system

The partners agreed on a quality assurance process regarding item writing, item reviewing (prior to trialing), trialing of items, psychometric analysis of item responses, and final item banking. This included setting up a Quality Assurance committee chaired by EAEVE and comprising two senior teachers from each partner VEE with academic expertise within the knowledge domains of the blueprint. The tasks of the QA committee are summarized in Table 2.

**Table 2:** Tasks of the quality assurance committee.

Ensuring an even distribution in scientific content in the item repository, across EAEVE’s Day One Competences and scientific areas of underpinning knowledge and skills
Feedback to partner institutions on the need for additional items within any scientific area that are lacking in the item repository
Guiding the training of representatives of the partner institutions in writing items and performing content analysis
Selecting items, in accordance with the blueprint prior to pilot testing
Making final decisions on which items to be placed in the repository or be resubmitted following the psychometric analysis of items
Regular control of items in the repository to ensure that their content is up to date, revised, and subsequently re-tested

A QA manual including guidelines, item templates for item writers, and a YouTube video on progress testing targeting students (27) were produced within the first 6 months of the project, and three online seminars on writing items for the VetRepos item repository were held during the first 12 months of the project. Later, two 2.5-day face-to-face workshops for item-writers and a short (private) online course (SPOC) on “Writing test items for measuring student progress” (28) were developed and launched during the project period to support teachers’ item writing.

**Figure 2:**
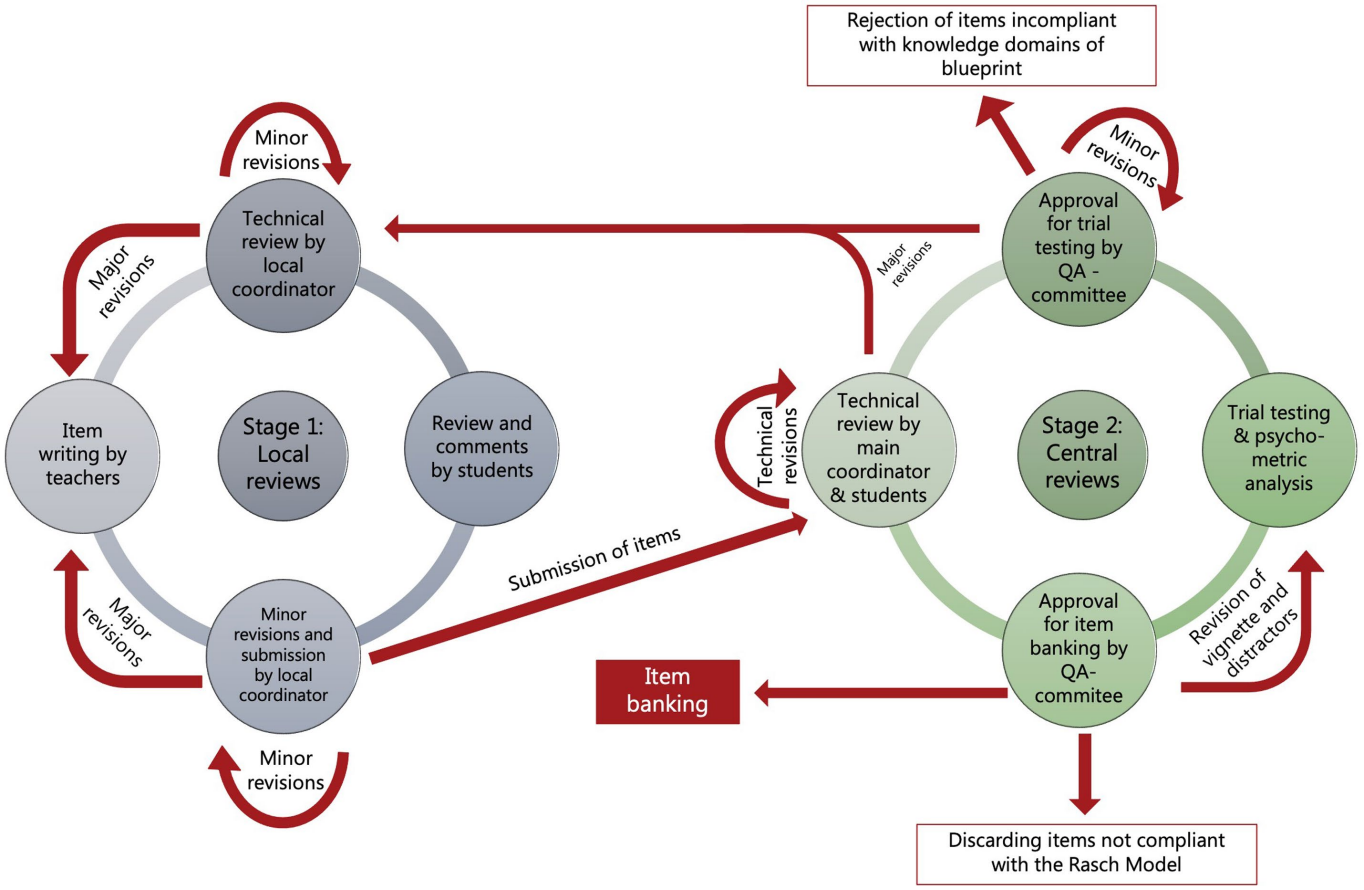
Schematic illustration of the organization of the quality assurance process for items.

The QA process was divided into three stages. The first stage was performed locally at each of the partner VEEs comprising guidance for teachers volunteering to item writing, and a peer-review of written items to check if they complied with the guidelines and requirements. It also included reviewing by 2–3 students, who commented on the items and register item response times. If the result of reviews necessitated a major revision of items, items were returned to the item writer. If the revision required was minor, the local project coordinator performed the necessary revision. Locally approved items were submitted to the project secretary for the (central) second stage QA process. Here, submitted items were reviewed again: first by the project secretariat for technical and language flaws and then by the QA committee for compliance with the blueprint and guidelines, including the decision on whether the academic content of the item targeted common Day 1 competencies of veterinary graduates. The QA committee approved items for trial-testing. The third stage of the QA process involved item trailing, psychometric analyses of item responses, and finally approval of items for item banking by the QA committee. All QA meetings were held as online sessions. Figure 2 illustrates the organization of the QA process.

### 2.5. Item writing

Initially, the partners agreed that each VEE should deliver approximately 300 items within the four subscales over the project period for QA and pretesting summing up to ≈1800 submitted items in total for trialing. It was expected that about a third of submitted items would have to be discarded in the review and trialing process due to content balancing and/or misfit to the psychometric model, thus ending up with ≈1,200 accepted items in the repository where each question in a polytomous item counts as one item.

Teachers at all universities were invited to write items within their veterinary competence domain after receiving the QA manual with the item writing guidelines and item templates. They were also invited to attend the online item writing seminars and the face-to-face workshops, and to watch the YouTube video on progress testing (27) and the SPOC on item writing (28).

### 2.6. Student recruitment and feedback

Voluntary veterinary students from all study years were recruited from the partner VEEs, which all together hold around 5,000 enrolled students. The total number of enrolled students varied among VEEs from slightly more than 500 to more than 2,000 students. Recruitment was done through poster campaigns, announcements on the intranet of VEEs and on student social media, direct emailing, and/or direct promotion at lectures. An initial campaign was launched in December 2020, followed by campaigns immediately before each trial test and one or two email reminders, while the tests were running. In addition, it was decided to hire student assistants who also helped with recruiting students into the project. For the last trial test, students from the another European university of veterinary medicine interested in the project were also invited to take part.

**Figure 3:**
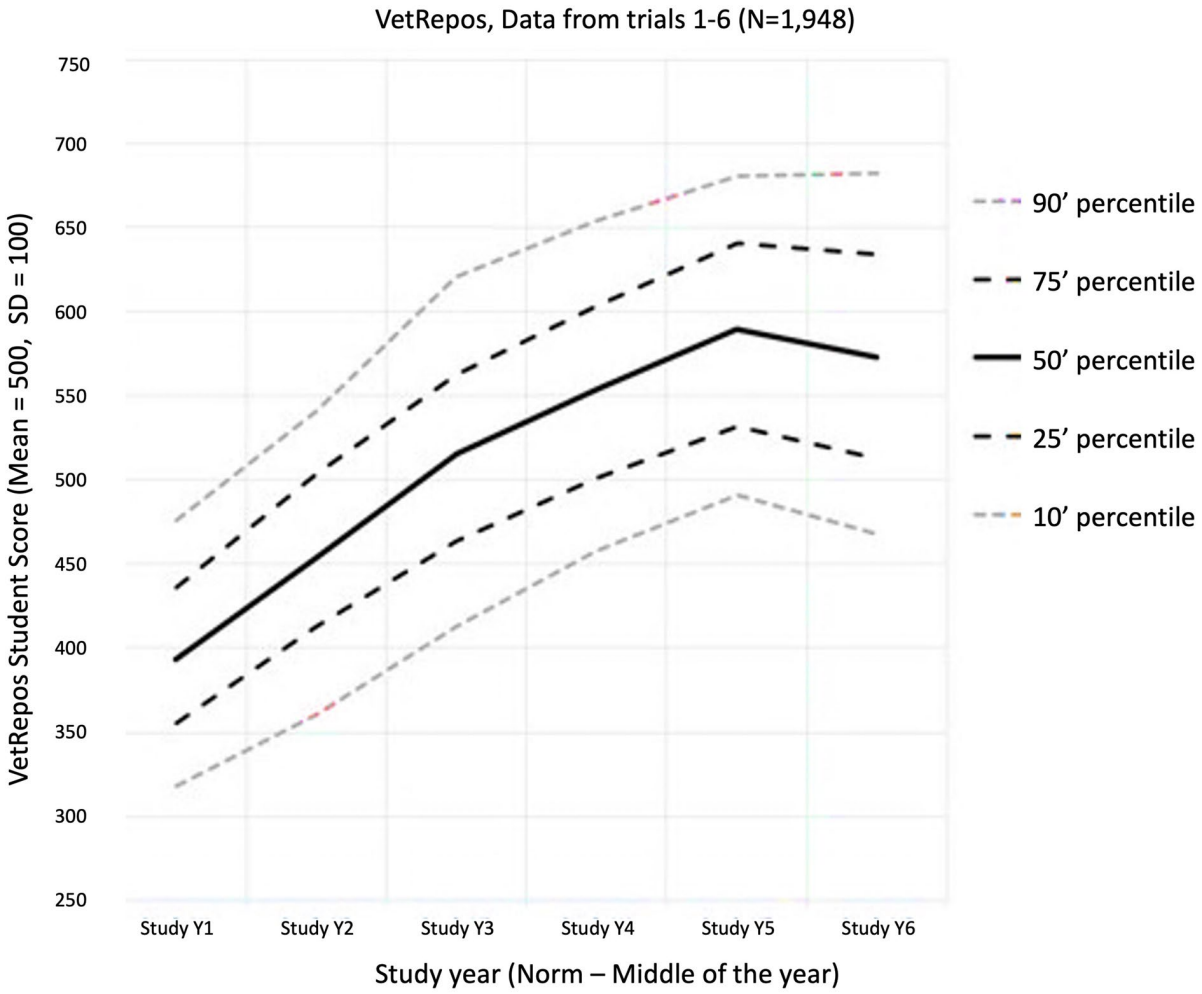
The measured progress (estimated growth curves, Aug. 2023). Distribution of student average scores within study year cohorts (x-axis) measured on the VetRepos scale (y-axis). The scale is a linear transformation where the Rasch scale reference point of 0 refers to the mean score 500 points with a standard deviation of 100.

Individual feedback to students on their test results was given in personal emails. The feedback was sent out after the psychometric analysis had been completed. The feedback consisted of a total score and subscale scores accompanied by a table of the average total scores and subscale scores for the Year 1 to 6 student cohorts, respectively, and an explanation of how the scores should be interpreted. Students were also supplied with a link to the latest “Estimated Growth Curve” of knowledge (see Figure 3). This allowed students to benchmark themselves to a common average. The scores and scale were a linear transformation of the Rasch logit scale for the model ranging between around 200 to 800, with a medium of 500 and standard deviation of 100. No feedback was given on the responses to individual items during or after the test, but a total “raw score” (number of correct answers/number of items in the test) was automatically given to the students at the end of the test.

### 2.7. Trialing of items

The item trialing was planned as a series of trial tests (2 per semester, 10 trials in total) to be conducted between the Spring semesters 2021 and 2023, as items were submitted and reviewed. Each trial test was designed to encompass 150–200 items with an estimated average response time of a maximum of 1.5 h. The Qualtrics^TM^ software (29) was chosen as the test platform as it was used as the common survey platform at one partner university and complied with EU GDPR regulations.

Following the initial trial test of the first items, we conducted an analysis of item responses, and we identified 28 items covering all subscales that exhibited an excellent fit with the Rasch Model, well-performing distractors, and substantial variation in both difficulty and content (subscales). Those 28 items were used as anchor items in the subsequent item trial tests and were included in all subsequent trial tests to link all items to the same coherent scale.

All trial tests were accessible for students for 4 weeks or until at least 300 complete responses were received. Students accessed the tests directly via URL links or QR codes provided in the recruitment emails and promotion campaigns. Students could pause their response activity and log out and log in to the test freely if done from the same computer and browser.

The item trial questionnaire was presented in five sections: (i) Welcome text including the project Privacy Policy (GDPR) to which test takers had to agree to continue, (ii) Seven background questions (university, study ID, study mail, username, gender, birth year and study year), (iii) Project background information including test subscales and disciplines; (iv) Test items subdivided into (a) anchor items; (b) classical MCQ and Matrix items, and (c) Cloze and other items, and (v) Conclusion, including a “free-text field” for students to give feedback on the test and the items. Items within a subsection were presented to individual students in random order.

Test responses were exported from Qualtrics as Excel files to a secure server at the Sensitive Information Facility at the University of Copenhagen (30) from where they were downloaded for psychometric analysis using the RUMM2030 software (31)_._

### 2.8. Psychometric analysis and item banking

As the established item repository must be able to support both linear and computer-adaptive progress testing, Rasch Modeling and analysis (20) were used for psychometric validation and calibration of items, including estimation of the difficulty of items. The method assumes the existence of one common construct, i.e., a common ability (a latent trait) that encompasses the knowledge and skills tested by the items. The model also places the item difficulty on the same scale as the student’s ability (scores). In the VetRepos project, this implies that the abilities of students from all study years and across participating VEEs have been assessed on the same unidimensional scale that fits the Rasch model. Hence, it is imperative to identify a scale that demonstrates reasonable consistency when evaluated across the VEEs, each of which has a different veterinary curriculum and mother language. Furthermore, this scale should be capable of effectively measuring academic progress from the first to the final year of the programs, even though the curricula traditionally exhibit significant variations between VEEs. It was therefore decided to adopt an investigative, but pragmatic approach, meaning that items had to fit the model to a reasonable extent, if not, they were discarded. If a more rigid approach was adopted, one could – beyond a reasonable doubt – reject any model. Though the applied model was based on an overall unidimensional scale, the items were categorized by their content into four dimensions or content areas that we call subscales (content areas, see Table 1).

The item analysis consisted of three steps for each item trial test: (i) Analysis of Item-fit with a particular focus on identifying and examining item fit residuals that numerically exceeded 2.5 logits, (ii) Analysis of Differential item functioning (DIF) by study year, gender, and university (country), and (iii) Analysis of Item Characteristic Curves (ICCs) and distractors with special attention given to investigating items with large fit residuals (poorly discriminating items) and items with very high item locations (highly difficult items).

Once the item analyses were completed, the results (estimated item locations, fit-residual values, and distractor curves) were presented to the QA committee for the purpose of deciding whether to approve or discard items. The results were discussed between the content experts of the committee and psychometricians. In some cases when the item’s data were near threshold values, and the content of the item was considered particularly important, the QA committee chose to disregard the recommendation based on item response analysis. However, in most cases, the QA committee followed the recommendation. In such instances, the QA committee opted to re-trial items after making minor alternations to the vignette or distractors.

### 2.9. GDPR and project privacy policy

The VetRepos project complies with the European General Data Protection Regulation (GDPR) and was approved and registered by the Research Data Management and GDPR office of the University of Copenhagen (Case no.: 514–0675/21–3000).

All students participating in the trial tests and associated questionnaires were at the start of each test and questionnaire – prior to giving their consent to this in order to participate in the test – informed about GDPR (including their right to “be forgotten” and have all data concerning them deleted) and the Private Policy of the project and the data processing and storing procedures.

## 3. Results

### 3.1. Item writing and reviewing

In total, 1,327 individual items were submitted from partner VEEs after the first stage review, including several items that were resubmitted after revision. The contribution of items varied between partners from 58 to more than 600 items.

The items comprised 1,211 classical (dichotomous) MCQ items, five polytomous MCQ items, 90 matrix items, 21 cloze items, and no (zero) numeric items. Apart from these items, six classical MQC items were supplied by the International Council for Veterinary Assessment from their pool of retired items from the North American Veterinary Licensing Exam (32). The polytomous MCQs, the matrix items, and the cloze items contained 625 separate questions (subitems), which were treated as individual item responses in the analysis. Hence, the total number of questions requiring a student response was 1,836 including all questions of all item formats.

Table 3 gives an overview of the number of submitted, reviewed, and rejected items including subitems within each of the subscales. It is worth noticing that over half of the submitted items were rejected prior to trialing. Most rejections occurred due to technical/item writing flaws and content redundancy before items were sent to the QA committee. The most frequent reason for rejection by the QA committee was noncompliance with the requirements defined in the Blueprint, meaning that the content of the item was considered to fall outside the scope of common veterinary Day One knowledge and skills. But it also included duplicate items and items where the correctness of the answer prompted debate.

**Table 3:** Number of submitted, QA committee reviewed, and banked items across subscales*.

	Total	Subscale 1	Subscale 2	Subscale 3	Subscale 4
Submitted	1,836 (100%)	1,066 (100%)	357 (100%)	409 (100%)	228 (100%)
Reviewed by QA – committee	1,342 (73%)	810 (76%)	248 (69%)	313 (77%)	179 (79%)
Accepted for trialing	961 (52%)	566 (53%)	195 (55%)	242 (59%)	151 (66%)
Banked	821 (45%)	470 (44%)	168 (47%)	203 (50%)	132 (58%)

*Items associated with more than one subscale are included in each of the associated subscales.

The most common technical/item writing flaws included overly long and/or complex response categories, implausible distractors, and unnecessary information in the vignette.

### 3.2. Student participation

Due to the COVID-19 situation at partner universities in 2020, the initiation of item trialing had to be postponed by 8 months. In total, six item trials were administered between December 2022 and June 2023, each 2–6 months apart. The trials were kept open between 4 and 12 weeks to obtain at least 300 responses, which was considered to be the minimum number of responses required for robust psychometric analyses. For the first two trial tests, enough responses were collected within 4 weeks, whereafter 8–12 weeks were needed for the last four trial tests.

1,119 individual students from all study years and all partner VEEs participated in one or more item trials. Several students did more than one item trial, but most students responded only to one trial (*n* = 702). Altogether, 1,948 usable test responses, were collected, ranging from 239 to 429 complete (and a few partial) responses per trial.

In Table 4, the distribution of student responses across VEEs and study years is listed. The data shows that the second-year student cohort constituted the largest group of respondents (41%) and that participation gradually declined with increasing years of study.

**Table 4:** Distribution of trial-test responses across student years and universities.

	Year 1	Year 2	Year 3	Year 4	Year 5	Year 6	Total
Uni1	58	64	77	46	43	38	**326**
Uni2	17	55	49	55	45	48	**269**
Uni3	54	53	76	87	60	34	**364**
Uni4	48	40	32	30	22	11	**183**
Uni5	14	33	46	32	32	28	**185**
Uni6	176	177	97	72	34	17	**573**
Uni7	0	16	13	9	6	4	**48**
Total	**367**	**438**	**390**	**331**	**242**	**180**	**1,948**

Students from all universities participated in all 7 trial-tests, except Uni 7, which students were invited in for trial-test 6, only.

### 3.3. Student comments

Students reported 273 comments in the trials. Most comments (*n* = 229) were reporting specific errors or ambiguities in items, e.g., language errors (*n* = 202), questioning the correctness of item content (*n* = 10), and identification of similarities between items (*n* = 7). A little less than 20% of comments related to more general aspects of the tests, such as the use and difficulty of the English language including medical English terminology (*n* = 35), and the setup of tests including item numbers and difficulty of the tests (*n* = 10). Finally, some students commented on their own general performance in the test or had other personal comments (*n* = 9).

### 3.4. Psychometric analyses

#### 3.4.1. Student and item distribution on the Rasch scale

An important part of the Rasch analysis is the estimation of item difficulty for all items (item location) and student ability (person location) on the same logit scale (from – to + infinity). The Rasch scale center (reference point) is 0 and it is defined by the location of the medium difficult item. All scores are related to the item’s difficulty distribution. The student’s ability (person location) is defined as equal to the item location where the student has 50% probability of finding the correct answer.

**Figure 4:**
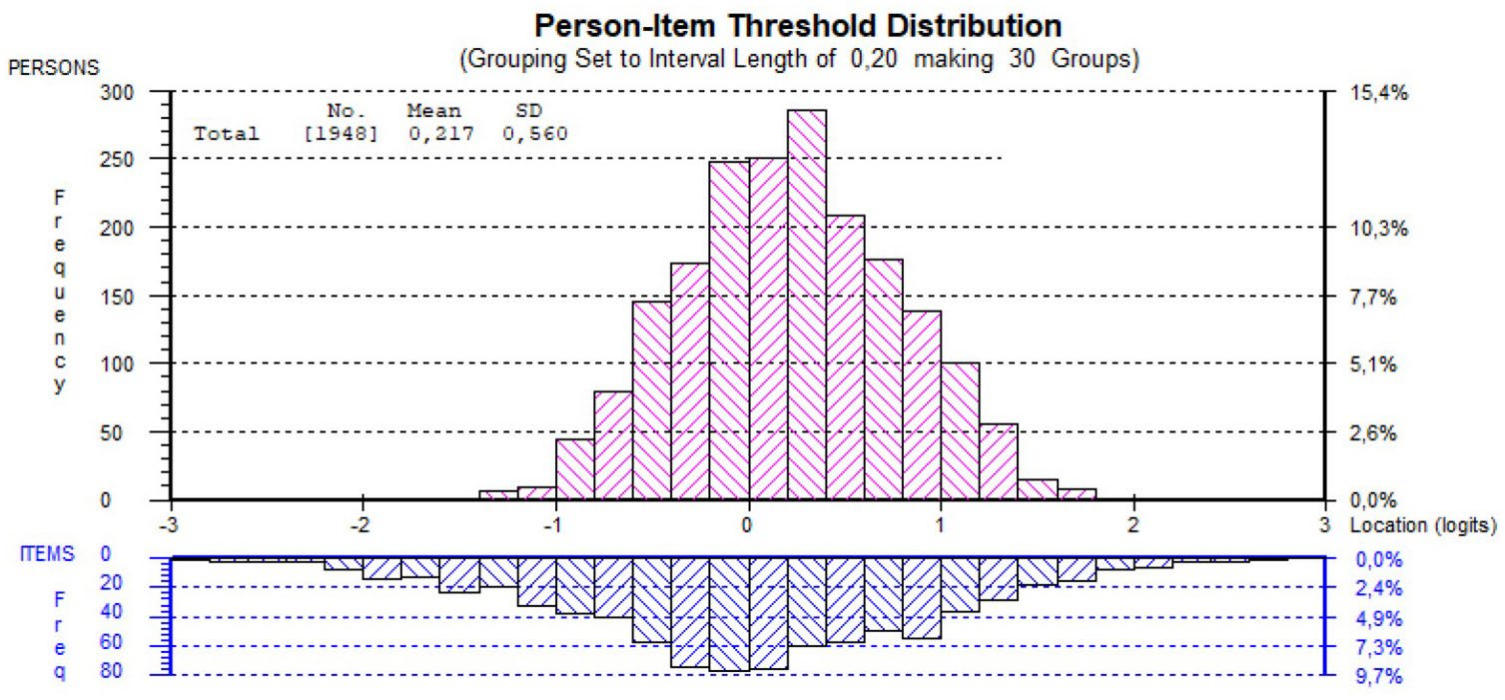
Rasch scale distribution of student ability (Red, *N* = 1,948) and item difficulty (Blue, *N* = 821) of the responses and banked items.

The distribution of student scores and item difficulties and their relation is shown in Figure 4. The figure demonstrates that there is an excellent match between the two distributions (good targeting) and that the item location distribution is wider on both sides than the student location distribution. That means that there are items that even the worst-performing student has a fair chance to get right and that there are items so challenging that even the best-performing student has less than 50% chance to get it right. In other words – the item bank contains appropriate challenges for all veterinary students.

#### 3.4.2. Rasch analyses of differential item functioning

The Rasch analysis was done six times, one for each trial. Each time the analysis included all previous data (both responses and previously accepted items). After analyzing the item fit residuals, conducting distractor analysis, and undergoing the QA item discarding process, 821 items remained in the accepted item pool.

In the Rasch model, it is assumed that the probability of a correct response is solely influenced by the student’s ability and the item’s difficulty. When equally skilled individuals from different groups (e.g., males vs. females) display significantly different probabilities of answering a specific item correctly, that item is deemed to exhibit Differential item functioning (DIF). As expected, some items, e.g., items with specific clinical content, exhibited DIF in relation to Study Year, and we expected DIF was also a possibility in relation to University.

##### 3.4.2.1. DIF for gender

**Figure 5:**
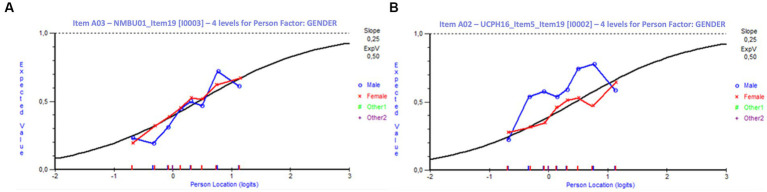
Differential item functioning curves for gender. **(A)** Item without DIF for gender. **(B)** Item with signs of DIF for gender. Item A02 **(B)** will favor moderately proficient males (blue curve) compared to equally proficient females (red curve). These males have a 10–20% higher probability of answering correctly.

Female students dominated the present study with 1,713 responses compared to 222 responses from male students and 13 from students of other genders. This distribution of responses reflects the general gender distribution in the participating VEEs. The analysis for DIF indicated that only one item exhibited significant DIF (at a 5% significance level) with respect to gender. A few additional items including one of the anchor items, showed signs of DIF, although not statistically significant. Since the population is heavily skewed, and most items apart from the anchor items were answered by approximately 300 students, the analysis of these items is based on limited data (anchor items were responded by all students in all trials, *n* = 1,948). Figure 5 shows DIF curves for gender for two anchor items, one without DIF and one with signs of DIF. The latter item favors moderately proficient males over similar proficient females. However, since it does not favor all students across the entire range of ability, there is no significant DIF on this item.

##### 3.4.2.2. DIF for university

The analysis indicates significant DIF (at the 5% level) for 52 items. For the majority (*n* = 33) of these, DIF is significant at all levels. The 52 items account for just over 6% of the total 821 items. It is therefore unlikely that the DIF has any significant influence on the total scores of students.

##### 3.4.2.3. DIF for study year

The DIF analysis for Study Year reveals that certain items functioned differently for first-year students compared to those with more experience. There are 37 items exhibiting significant DIF (at the 5% level); just under half of these show DIF at all ability levels. Thus, the DIF for Study Year concerns approximately 4% of items, which can be considered a relatively modest extent, and it does not significantly influence the totals scores of students and thereby the measurement of their general growth in proficiency as they progress through the curriculum (see “Student scores and progress on the VetRepos scale”).

#### 3.4.3. Student scores and progress on the VetRepos scale

The Rasch logit scale is not intended to be used for feedback to students and teachers, as it includes negative values, and the reference point is item difficulty – not student ability. Therefore, with inspiration from international large-scale assessments (33), the scores on the Rasch logit scale were, linearly transformed (34) to a scale, where the Rasch scale reference point, equivalent to the medium item difficulty level at which students have a 50% chance of getting the correct answer, refers to the mean score 500 points with a standard deviation of 100.

The growth in proficiency of the students can be measured by comparing scores across study years, which are illustrated in Figure 3. The progress from year to year is largest among the best-performing students, and the annual progress seems to be decreasing over the year. The overall annual progress of students participating in the study is estimated to be 47 points on the test scale.

### 3.5. Item banking

Based on the psychometric analyses, including visual analysis and discussions of item characteristics and distractor curves for items that did not fit the used model, the QA committee approved 86% of the trialed items for banking. A few items with a marginal fit that the committee regarded as academically important were temporarily accepted, some after revision – and then re-trialed. However, most of the re-trialed items were finally discarded due to not fitting in with the model.

**Figure 6:**
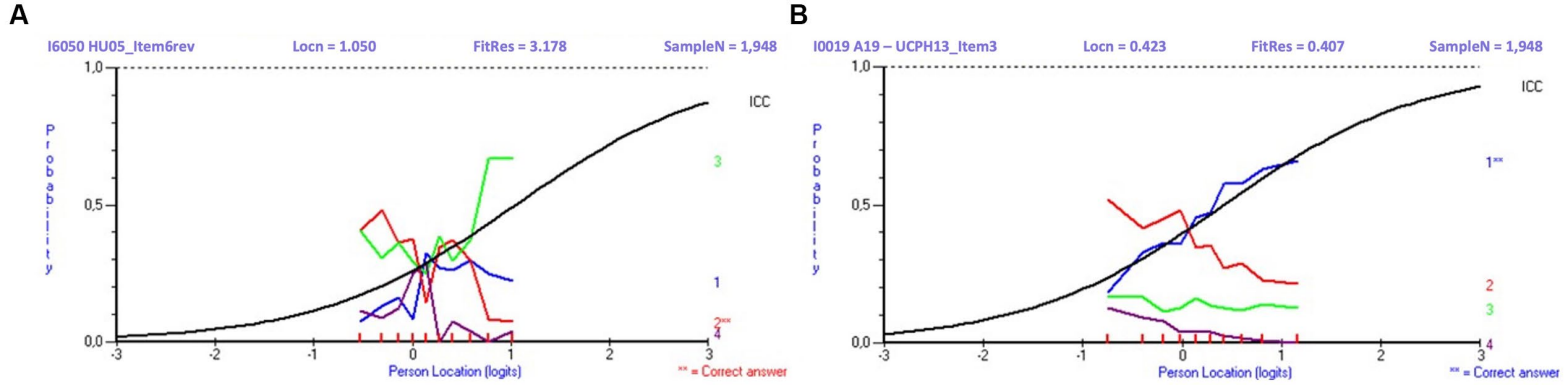
Distractor curves for a poorly **(A)** and well-performing item **(B)**. The response curve for Item **A** shows that the more skilled the student is, the less likely the student is to choose the correct answer (red curve), but instead is more likely to choose a certain distractor (green curve). The response curve for Item **B** shows the more skilled the student is, the more likely the student is to choose the correct answer (blue curve). Legends: Locn, item location on Rasch Scale (difficulty level); FitRes, item Fit Residual; SampleN, sample size.

Overview of inputs (tested items and student responses) and outcomes (number of banked and discarded items) of the Rasch analyses and QA process performed after each of the six trial tests is described in Table 5. Examples of distractor curves, used by the psychometrician and the QA committee in their visual analysis of items, are shown in Figure 6.

**Table 5:** Overview of number of responses and items and outcomes (number of banked and discarded items) of the Rasch analyses performed after each of the 6 trial tests.

	Number of student responses		Number of items (number discarded items in brackets)								
	In specific trial	In Rasch analysis	In Rasch analysis	Anchor items	Trial1	Trial2	Trial3	Trial4	Trial5	Trial6	Approved (banked)
Trial1	429	429	153	–	153 (31)						122
Trial2	239	668	297	28		144 (21)					245
Trial3	297	965	492	28			195 (22)				418
Trial4	421	1,386	713	28				221 (33)			606
Trial5	274	1,660	812	28					99 (17)		688
Trial6	288	1,948	961	28						149 (16)	821

The final numbers of banked items within the four subscales are listed in Table 3, and the final distribution of banked among the 34 EAEVE veterinary disciplines is shown in Table 6.

**Table 6:** Distribution of banked items across disciplines*.

Test subscales	EAEVE subject area	Banked items	Aim according to blueprint
Basic Sciences (Subscale 1)	1. Anatomy, histology and embryology	58	35
	2. Physiology	40	35
	3. Biochemistry	21	35
	4. General-, population- and molecular genetics	81	35
	5. Pharmacology, pharmacy and pharmacotherapy	54	35
	6. Pathology	29	35
	7. Toxicology	20	35
	8. Parasitology	22	35
	9. Microbiology	122	35
	10. Immunology	23	35
	11. Epidemiology	20	35
	12. Information literacy and data management	14	35
	13. Professional ethics and communication	6	35
	14. Animal health economics and practice management	0	35
	15. Animal ethology	9	35
	16. Animal welfare	3	35
	17. Animal nutrition	9	35
Clinical Sciences – Companion Animal & Equine (Subscale 2) and Clinical Sciences – Production Animals, including animal production subjects (Subscale 3)	18. Obstetrics, reproduction and reproductive disorders (in “production animals” + “companion animals and equines”)		44 (24 + 20)
	19. Diagnostic pathology (in “production animals” + “companion animals and equines”)	29 (11 + 18)	44 (24 + 20)
	20. Medicine (in “production animals” + “companion animals and equines”)	36 (25 + 14)^1^	44 (24 + 20)
	21. Surgery (in “production animals” + “companion animals and equines”)	163 (62 + 105)^2^	44 (24 + 20)
	22. Anesthesiology (in “production animals” + “companion animals and equines”)	36 (36 + 6)^3^	44 (24 + 20)
	23. Clinical practical training in common animal species in “production animals” or “companion animals and equines”	13 (11 + 2)	44 (24 + 20)
	24. Preventive medicine (in “production animals” + “companion animals and equines”)	31 (13 + 21)^4^	44 (24 + 20)
	25. Diagnostic imaging (in “production animals” + “companion animals and equines”)	7 (2 + 6)^5^	44 (24 + 20)
	26. Therapy (in “production animals” or “companion animals and equines”)	17 (17 + 0)	44 (24 + 20)
	27. Propaedeutics (in “production animals” + “companion animals and equines”)	6 (4 + 2)	44 (24 + 20)
	28. Animal production, including breeding, husbandry and economics	31	20
	29. Herd health management	15	20
Food Safety & Quality, Public Health and One Health Concepts (Subscale 4)	30. Veterinary legislation including official controls, regulatory veterinary services, forensic veterinary medicine and certification	26	24
	31. Control of food, feed and animal by-products	32	24
	32. Zoonoses	26	24
	33. Food hygiene and food microbiology	57	24
	34. Food Technology	20	24

* Items associated with more than one discipline are included in each of the associated disciplines. ^1,2,3,4,5^One or more of the items within the discipline is associated with both “Production animals” (subscale 3) and “Companion Animals and Equines” (subscale 2) and are therefore included in both subscales.

## 4. Discussion

### 4.1. Student participation and feedback to and from students

Around 20% of the students enrolled at the partner VEEs responded to one or more trials, which is comparable to the response rate in a similar study among eight European medical schools (35). However, the trialing process disclosed that students’ engagement declined as the project developed. It became more difficult to get students to respond to tests within the originally planned 4-week period, and just around 10% of the responding students answered four or more trial tests.

A few responses also indicated that not all students engaged properly in the test, as, e.g., the response pattern for the MCQs in the test revealed that one of the four response categories was mainly chosen, or – if a student for some reasons – had answered the same test item twice and the two sets of responses were very different (such duplicate responses were deleted from the analyses).

The decline in engagement may be explained by the fact that the veterinary curricula are generally overloaded with compulsory learning activities including summative exams (36), so additional voluntary and unfamiliar learning activities are therefore likely to be rejected if students do not feel they benefit from them (37). Statements from students at a curriculum board meeting at a partner VEE, where future implementation of progress testing was discussed, pointed explicitly in this direction. Here students specifically articulated that participation in future formative progress testing should be voluntary, as the workload and number of study activities were already very high (38).

Most students only participated in one trial test, even though video statements from students who participated in more tests confirm that a group of students find this form of testing very exciting and motivating (39). The students’ comments from the trials indicate that the test time mattered, and too-long tests discouraged students. Some students were also discouraged by what they perceived as difficult items. A mismatch between item difficulty and student ability is as earlier mentioned an embedded issue in linear progress tests because many first-year students and low-performing students will find most item content unknown. The use of the English language added to the discomfort with the tests, according to some students. Thirty-five comments of 273 in total on the English language indicate the language is relevant. As the majority of students are non-native English speakers, English language skills may have an impact on test scores and on student motivation, even though items were to be written in an unpretentious and clear common English language using correct Latin/Greek based medical terms. Authors assume that English skills among their students are relatively high and comparable across the VEEs. This may not be the case in the future, if more VEEs join the collaboration. The influence of the language should therefore be analyzed in more detail in the future. In this regard, Phisalprapa et al. (40) investigated the influence of English language multiple choice questions in an exam and concluded that their use may reduce test scores, especially for borderline students. However, Rice et al. (35) showed in their study regarding the development of a computer adaptive progress test among eight European medical schools with written items written in English that stable estimates of ability, low standard errors of measurement and high test reliability can be obtained. In this study, 30% of the students were native English speaking, while more 99% of the non-English native speaking students reported themselves as proficient in English.

Educational research indicates that computer-adaptive testing, which the VetRepos item repository is prepared for, can be more efficient and student-friendly than linear testing. By computer-adaptive testing valid and reliable measurements of students’ abilities can be reached with considerably fewer items (18, 41), and item difficulty and student ability are better matched. This is likely to affect students’ engagement in progress testing positively (17, 42), which is important for successful implementation of progress testing if tests are voluntary and only serve formative purposes. The validity of the result is dependent on the students having worked attentively and done their best (43).

Meaningful and comprehensive feedback is an essential prerequisite and a major motivational factor in progress testing that positively affects students’ learning behavior (3, 5, 44). It is also the major reason for students to engage in progress testing if tests are implemented as voluntary and pure formative tests.

It has been said that “From our students’ point of view, assessment always defines the actual curriculum” (45), and that students are “guided more by the nature and content of tests than.

by curriculum descriptions of learning outcomes” (46), statements that are supported by educational research (47, 48). Hence, when implementing formative progress testing into a curriculum it is of utmost importance that the VEE provides a framework in which the tests are perceived as an important part of the curriculum by students and teachers, in line with other assessments within the curriculum. Otherwise, it is questionable whether it can fulfill the purpose of supporting changes in study behavior toward deeper and long-lasting learning.

Institutions that have implemented progress testing use different tools apart from appropriate feedback to ensure student participation and engagement. In most institutions, the tests are obligatory, either as a compulsory formative assessment activity or as part of their summative exam system (5, 10, 49). Some institutions keep tests voluntary but motivate students to engage in the tests by giving credits for participating (25). In some Dutch medical schools, summative progress testing has replaced traditional theoretical summative course exams (49), thus tests are not adding to the number of obligatory study activities.

In the present project, the feedback to students was given 4–8 weeks after the closing of the trials, and for most students, the feedback did not contain progress information as less than half of students responded to more than one test. Therefore, the feedback should be regarded as suboptimal compared to feedback from normal progress testing, where feedback is given immediately after the closing of the test and students are able to see their progress from the second test and maybe compare themselves to the average student of the same study year (8, 50). However, in the present study, we reached enough responses to calibrate and bank items despite the decline in student engagement over the project period and suboptimal feedback. But the experiences point to a very important issue if implementing progress testing for purely formative purposes: How to ensure that students, including poor-performing students, engage in progress testing, so both most students benefit from progress testing, and the VEE gets value for manpower and money spent on it?

Our experiences from the present study indicate that most involved VEEs who plan to implement progress testing in their curricula will adhere to the formative use of the progress test but make participating compulsory.

### 4.2. Item writing and reviewing

The rejection rate of 52% of the submitted items prior to item trialing is high compared to a similar project between medical educational establishments (35), where half of the involved establishments had extensive experience with progress testing in their educations. In the present project, only one of the partners had extensive and present experience with item writing for progress testing and with rigorous and standardized item review process (25), which may explain the high rejection rate.

The experiences from the project show that not all VEEs were able to deliver the intended number of items. The results support that writing good items for progress testing is a difficult task requiring training (26), that a rigorous review of items by peers, students, and experts is needed to ensure item quality (3, 51). At the start of the project, only classical MCQs were submitted, presumably because this is the most common item format used by the VEEs. However, as the project went on and more teachers participated in the item writing training events, MC-matrix and MC-cloze items were generated, some with new content, others transformed from earlier rejected classical MCQs into one of these formats.

The rate of items rejected by the QA committee due to noncompliance with the blueprint remained relatively constant throughout the project. This includes items whose content was condemned to be outside the common veterinary Day 1 competencies. The QA committee continued to make minor revisions to a substantial part of the items prior to item trailing, e.g., general English language, English medical, and Greek/Latin medical terms, and identified a few but increasing number of “doublet items” with almost identical content to already banked items. Hence, the experiences emphasize the importance of having an expert group as the last stage in a rigorous review of items.

Despite the review process, students participating in the trials identified items, albeit only a few, with spelling errors, items with language ambiguities, and items with similar content to other items. Some of the errors were due to manual flaws when copy-pasting from the QA-committee master file to the used Qualtrics test software, but others were mistakes that none of the previous reviewers had spotted. This may be explained by the large number of items that had to be reviewed over a relatively short time to fulfill the project aim, but again supporting the need for a rigorous review. Rigorous review of items is time and resource-demanding even though engagement in collaborative progress testing may reduce this effort considerably. Establishments that plan to implement progress testing need to prioritize this task if the calibrated item bank is to be maintained and regularly updated.

### 4.3. Test model and psychometric analyses

The applied test model has been able to deliver a reasonably reliable measure of students’ progress from the first to last trial test if the students have done their best. It has also been able to measure the growth in knowledge and skills within the student cohorts from the first to the last study year, showing a trend of increasing abilities from study year 1 to year 5, but a slight decline in the final year (see Figure 4). Similar growth curves with negative trends during the last study year have been reported by others in medical education (35), including veterinary medicine (25), and is likely to be associated with the educational focus of the final year on providing students hands-on clinical experience and practical training.

The psychometric analyses revealed, as anticipated beforehand that some items show signs of DIF for University and Study Year. Obviously, some level of DIF for Study Year must exist in progress testing targeting a whole academic curriculum, as the curricular focus normally changes from basic sciences within the first years to clinical and veterinary public health-related teaching, which (hopefully) leads to progress in the abilities of older students but probably also to some loss in their knowledge and skills within basic sciences compared to students from the first study years.

In large international progress test collaborations, the curricula will differ and national focus on specific veterinary knowledge domains may be reflected in the number of items and/or item content and students’ abilities associated with such domains, which may lead to DIF. A well-balanced item repository with comparable numbers of items from all user universities may reduce the significance of DIF on test results.

However, as the extent of items showing DIF is limited and the magnitude of their DIF is relatively small, the effect of the DIF on individual test scores is likely to be undetectable, or at least negligible in a low-stake test setting, which is the intention with the VetRepos item repository. If, however, a significant number of items show substantial DIF, especially in a high-stakes test, it can lead to unfairness and bias in the scores for certain groups of test-takers. Detecting and addressing DIF is therefore crucial (52), also in relation to low-stakes tests, to ensure proper validity and calibration of the item databank over time. This includes the VetRepos item repository.

The main purpose of the VetRepos project is to supply VEEs with a tool that allows the students to monitor their progress and thereby motivate them for stable longitudinal learning. However, the individual VEEs may of course also use the aggregated progress data to shed light on the outcomes of their provided teaching and curriculum, e.g., comparing the progress of students enrolled on different trackings within their veterinary program. The aggregated progress data may also be used for benchmarking (5, 11, 13). However, many factors influence students’ progress, and aggregated data should always be handled with care. This certainly includes attempts to compare progress data from individual universities. The partners of the VetRepos project have made the deliberate decision not to pursue the possibility of direct comparison between partner VEEs, but only to reveal data that allows for benchmarking to a “project average.”

### 4.4. The item repository and distribution of banked items on subscales and disciplines

The banked 821 calibrated items cover all EAEVE veterinary science knowledge domains. As several items cover more than one knowledge domain, the distribution of items can be said to fulfill the anticipated distribution of 50% of items within Basic Veterinary Science subjects, 20% within the respective Veterinary Clinical Science domains (companion animals and equines, and production animals including some Animal Science subjects) and 10% within the area of Food Safety, Veterinary Health and One-Health. The total number of banked items was less than anticipated at the project start, as the delay of the initiation of trial testing and a limited number of particularly clinical science items submitted hampered the outcome. It is our experience that multiple-choice items within clinical sciences addressing clinical reasoning were challenging for teachers to write. As a consequence, a few clinical disciplines (EAEVE subject areas) are underrepresented in the item repository, e.g., Clinical Practical Training, Propaedeutics, and Diagnostic Imaging. Also, a few basic science disciplines including animal ethology and welfare, and subjects addressing soft skills such as ethics, communication, and general information literacy are also underrepresented in the databank. Our experiences from the review process indicate that items targeting these subjects were also challenging for teachers to write, as many were rejected in the review process due to technical reasons. The future maintenance and development of the VetRepos repository will include production and trialing of more items covering these disciplines. Trialing of new items can be done as an integrated routine of ordinary progress testing.

Nevertheless, the number of validated and calibrated items in the repository is adequate for the initial implementation of progress testing in veterinary education in a setting, where VEEs maintain and develop the item repository as a collaborative effort.

## 5. Conclusion

During the last transnational meeting of the VetRepos project, it was concluded that the project goals were achieved. We now have 821 validated items stored in our item bank, which is sufficient to support adaptive progress testing in European VEEs including providing useful information about performance within the major veterinary science domains of the blueprint. We have also around 320 additional items in the review process, awaiting trialing and subsequent banking of approved items. A proven QA system is present both locally at the VEEs as well as centrally at the project management level. In addition, diverse training materials, such as a SPOC (28), a YouTube tutorial (27) and other materials (53), are freely accessible for interested VEEs.

An advanced assessment platform for implementation of computer adaptive progress testing (QuizOne) (35, 54) at the VEEs is being adapted and tested, so it embraces all the VetRepos Item formats. Since there is ample evidence that progress testing can stimulate longitudinal and lasting learning by supplying students with frequent individual feedback, all the VetRepos partnership VEEs, but one, has agreed to continue this project by contributing new items to the item bank and implementing adaptive progress testing in their curricula using the same test blueprint. The present VetRepos partners anticipate that other VEEs will join the collaboration in the future. In this way, progress testing based on items that have been produced, reviewed and psychometrically calibrated across veterinary establishments, may become a common educational tool in veterinary education and may allow future benchmarking toward a common EAEVE standard for the collaborating VEEs.

## Data availability statement

The datasets presented in this article are not readily available because the item repository data can only be made available for partners of the VetRepos project. The students’ data and their answers cannot be disclosed due to GDPR. Requests to access the datasets should be directed to T.vanHaeften@uu.nl.

## Ethics statement

The studies involving humans were approved by Research Data Management and GDPR office of the University of Copenhagen (Case no.: 514–0675/21–3000). The studies were conducted in accordance with the local legislation and institutional requirements. The participants provided their written informed consent to participate in this study.

## Author contributions

ES: Writing – review & editing, Funding acquisition, Writing – original draft, Investigation, Methodology. TH: Funding acquisition, Writing – original draft, Writing – review & editing, Investigation, Methodology. JW: Formal analysis, Funding acquisition, Investigation, Writing – review & editing, Methodology. AI: Funding acquisition, Writing – review & editing. JP: Funding acquisition, Writing – review & editing. CP: Writing – review & editing. PL: Funding acquisition, Writing – review & editing. PH: Funding acquisition, Investigation, Project–administration, Writing – original draft, Writing – review & editing, Formal analysis, Methodology.
